# A Rhodopsin-Like Gene May Be Associated With the Light-Sensitivity of Adult Pacific Oyster *Crassostrea gigas*

**DOI:** 10.3389/fphys.2018.00221

**Published:** 2018-03-19

**Authors:** Changlu Wu, Qiuyun Jiang, Lei Wei, Zhongqiang Cai, Jun Chen, Wenchao Yu, Cheng He, Jiao Wang, Wen Guo, Xiaotong Wang

**Affiliations:** ^1^School of Agriculture, Ludong University, Yantai, China; ^2^Changdao Enhancement and Experiment Station, Chinese Academy of Fishery Sciences, Changdao, China; ^3^Center for Mollusc Study and Development, Marine Biology Institute of Shandong Province, Qingdao, China

**Keywords:** adult oyster, light-sensitivity, RNAi, mantle, rhodopsin-like superfamily member gene

## Abstract

Light-sensitivity is important for mollusc survival, as it plays a vital role in reproduction and predator avoidance. Light-sensitivity has been demonstrated in the adult Pacific oyster *Crassostrea gigas*, but the genes associated with light-sensitivity remain unclear. In the present study, we designed experiments to identify the genes associated with light-sensitivity in adult oysters. First, we assessed the Pacific oyster genome and identified 368 genes annotated with the terms associated with light-sensitivity. Second, the function of the four rhodopsin-like superfamily member genes was tested by using RNAi. The results showed that the highest level of mRNA expression of the vision-related genes was in the mantle; however, this finding is not true for all oyster genes. Interestingly, we also found four rhodopsin-like superfamily member genes expressed at an very high level in the mantle tissue. In the RNAi experiment, when one of rhodopsin-like superfamily member genes (CGI_1001253) was inhibited, the light-sensitivity capacity of the injected oysters was significantly reduced, suggesting that CGI_10012534 may be associated with light-sensitivity in the adult Pacific oyster.

## Introduction

Until recently, four classes of molluscs (Gastropoda, Bivalvia, Polyplacophora, and Cephalopoda) have been demonstrated as possessing light sensitive organs (Serb and Eernisse, 2008). The light structure, light type, and light functions of scallops (Land, 1965; Barber et al., 1967; Malkowsky and Jochum, 2015), chitons (Toomey et al., 2002), snails (Morton, 2000), slug (Morton, 2000), and nautilus (Kobak and Nowacki, 2007) have been extensively studied. Light-sensitive organs play an important role in reproduction and predator avoidance for the above molluscs (Wu et al., 2015). Eyes from molluscan lineages could be used to study convergence and parallel patterns of eye evolution (Nilsson and Kelber, 2007; Serb and Eernisse, 2008). However, little is known about the molluscan eyes below the molecular level.

Light-sensitivity in oyster larvae has previously been confirmed (Magalhães et al., 2014; Xu et al., 2015; Wheeler et al., 2017). After the occurrence of eyespot, the oyster larvae use their left shell to fix on the surface of rocks and other solid surfaces, and they no longer move after attachment. Although light-sensitivity does not significantly impact the movement of adult oysters, it may play an important role in the growth, reproduction, living habits, and anti-predatory behavior of these molluscs. In a previous study, we demonstrated that adult oysters are light-sensitive (Wu et al., 2015), but the genes associated with light-sensitivity remain uncertain. RNA interference (RNAi) is a transcriptional gene-silencing phenomenon mediated by double-stranded RNA (dsRNA), which can specifically silence target genes (Joga et al., 2016). Currently, RNAi has been widely used in zebrafish (Acosta et al., 2005; Chang and Nie, 2008), sea squirt, prawns (Aflalo and Sagi, 2014; Lezer et al., 2015), and other aquatic organisms. Pacific oyster was the first species of which the gene function was studied by RNAi among bivalves (Fabioux et al., 2009; Choi et al., 2013; Wang et al., 2013; Bo et al., 2014). After the completion of the whole genome sequencing of the Pacific oyster *Crassostrea gigas* (Zhang et al., 2012), RNAi technology was increasingly used in the oyster (Choi et al., 2013; Wang et al., 2013; Bo et al., 2014).

Rhodopsin, also known as the “visual purple,” is a molecular complex, consisting of a vitamin A-derived retinal chromophore, 11-cis-retinal, covalently bound to a seven transmembrane domain protein moiety (Van Hazel et al., 2016) and the largest class of G protein-coupled receptors (family A, subsequently referred to as GPCR-A) (Bryson-Richardson et al., 2004). Rhodopsin is a major component of rod photoreceptors, which are photosensitive, even under extremely weak light conditions (Hargrave and McDowell, 1992). The light-activated rhodopsin is the GPCR catalyzing the exchange of GDP for GTP on the heterotrimeric G protein transducin (Gao et al., 2017).

In the present study, we analyzed the vision-related genes in the Pacific oyster genome (Zhang et al., 2012) and studied the functions of rhodopsin-like superfamily member genes using RNAi methods combined with real-time fluorescence quantitative PCR.

## Materials and methods

### Animal preparation

*Crassostrea gigas* (shell height 70–90 mm) were obtained from Yantai, Shandong Province, China (121.39°E, 37.54°N) and maintained at the experiment station of the School of Agriculture, Ludong University. The oysters were acclimated in an aquarium tank (80 × 40 × 40 cm, length × width × height) supplied with filtered seawater at ambient temperature (16 ± 1°C) and salinity (30‰). The oysters were fed with microalgae *Isochrysis galbana* (5.0 × 10^5^ cell/mL) daily and allowed to acclimate for 1 week.

### Analysis of mRNA expression in vision-related genes

Vision-related genes were selected from the oyster gene set (Zhang et al., 2012) by examining the functional annotation of all oyster genes separately. Based on the oyster transcriptome data (Zhang et al., 2012), the mRNA expression pattern of these genes was analyzed at 37 different development stages (eggs and larvae were sampled from the mass spawning of 51 females and 1 male from the family “G3”) and in eight different organs (mantle, gill, adductor muscle, digestive gland, hemocyte, labial palp, and female gonad were obtained from one female oyster, male gonad from F1 offspring of family “G3”). In addition, we also assessed the genes specifically expressed in the mantle.

### The siRNA design and synthesis

According to the sequences of four rhodopsin-like superfamily member genes (CGI_10012534; CGI_10007162; CGI_10008927; CGI_10013409), two target siRNAs were designed and synthetized by Sangon Biotech (Shanghai) for each gene. The RNA interference locations for CGI_10012534 were labeled as 57TP and 59TP; those for CGI_10007162 757TP and 539TP; those for CGI_10008927 43TP and 31TP; those for CGI_10013409 76TP and 99TP. All the following siRNA sequences were used in this experiment (Table 1).

**Table 1:** The siRNA sequences in this experiment.

**Genes**	**Target Points**	**Sense (5′-3′)**	**Antisense (5′-3′)**
CGI_10012534	59TP	5′ GGUCCUCACAUUUGCUUAUTT 3′	5′ AUAAGCAAAUGUGAGGACCTT 3′
	57TP	5′ GGAGAGAAAUGGAGAAUAUTT 3′	5′ AUAUUCUCCAUUUCUCUCCTT 3′
CGI_10007162	757TP	5′ GUGCCGUAAUUUGUAAUAATT 3′	5′ UUAUUACAAAUUACGGCACTT 3′
	539TP	5′ CCGGUUCUUUACUCAAUAUTT 3′	5′ AUAUUGAGUAAAGAACCGGTT 3′
CGI_10008927	43TP	5′ CGGUCAACGUUCACAAUAUTT 3′	5′ AUAUUGUGAACGUUGACCGTT 3′
	31TP	5′ CCGUGCUUCAAUUUGUUUATT 3′	5′ UAAACAAAUUGAAGCACGGTT 3′
CGI_10013409	76TP	5′ GUGUGUACAUCUCCAUUAUTT 3′	5′ AUAAUGGAGAUGUACACACTT 3′
	99TP	5′ GGGAUGUGUUAGAUAUUGUTT 3′	5′ ACAAUAUCUAACACAUCCCTT 3′

### Flashlight experiments

Twenty-four hours prior to the start of the experiment, the oysters were randomly placed, one at a time, into a small transparent-glass aquarium (30 × 20 × 15 cm, length × width × height, respectively) through which air was continually pumped. When the oyster shells opened, a LED flashlight (light intensity was 5,000 Lux) was used to shine light onto the oysters' mantle and turned off after 40 s (Wu et al., 2015). After repeating 10 times, the light-sensitivity ability value of each oyster was calculated.light-sensitivitylight-sensitivitylight-sensitivity

The oysters responding to light were classified into three types that were all regarded as light-sensitivity: (I) individuals not only opening their shells wider after turning the light on but also closing their shells after turning the light off, (II) individuals only opening their shells wider after turning the light on, and (III) individuals only closing their shells after turning the light off (Wu et al., 2015). The light-sensitivity ability value was calculated as below: The light-sensitivity ability value = number of light-sensitivity before interference/number of illumination before interference.light-sensitivity When the light-sensitivity ability value reached to 0.8 or more, the tested oyster was regarded as having the light-sensitivity ability. At last, 48 adult oysters with the light-sensitivity ability were selected for RNAi experiments for each gene.

### RNAi experiment

To open the oyster shell without hurting the animal, all oysters were anesthetized in 8% MgSO_4_ seawater solution (Wang et al., 2011) (7:00 p.m., first day). After 12 h (7:00 a.m., second day), the shells of the oysters were opened. Two siRNAs of each gene were dissolved in PBS buffer for adductor muscle injection; oysters in the experimental groups were injected with the following treatments: 5 μg siRNA + 100 μl PBS, 10 μg siRNA + 100 μl PBS, or 15 μg siRNA + 100 μl PBS; the adductor muscle tissues of the oysters in control group were injected with 100 μl of PBS. After another 12 h (7:00 p.m., second day), these oysters were placed into a small transparent-glass aquarium, and flashlight experiments were conducted (repeated 10 times) at the opening of the oysters' shells to observe the responses of oysters to the light.

### Real-time quantitative PCR experiments

Three days after siRNA injection (7:00 a.m., fourth day), all oysters were sacrificed, and their in-mantle (inner mantle) and out-mantle (outer mantle) tissues were sampled. Total RNAs were extracted from the in-mantle and out-mantle tissues and reverse transcribed into cDNA for real-time quantitative PCR experiments. The mRNA expression levels were identified using the Trans Start Green qPCR Super Mix UDG Kit (TransGen) and analyzed by the 2^−ΔΔCT^ method described previously (Livak and Schmittgen, 2001; Bustin et al., 2009). The primers of four target genes and the reference gene (Cg-ef1α; Huvet et al., 2015) used in the real-time quantitative PCR experiment are listed in Table 2.

**Table 2:** The primers used in qPCR experiment.

**Primers**	**Sequence (5′ → 3′)**	**Application**
CGI_10012534F	GTGCAGGCCGTCATACTCTA	The forward primer for CGI_10012534
CGI_10012534R	CGCTGTCACCACCAATAC	The reverse primer for CGI_10012534
CGI_10007162F	TGTTGGCGTTCGCTCTGA	The forward primer for CGI_10007162
CGI_10007162R	CGTAATGGTCGTGTCTGC	The reverse primer for CGI_10007162
CGI_10008927F	GTGCAGGCCGTCATACTCTA	The forward primer for CGI_10008927
CGI_10008927R	CGCTGTCACCACCAATAC	The reverse primer for CGI_10008927
CGI_10013409F	TGTTAGATATTGTGGCGCTTTG	The forward primer for CGI_10013409
CGI_10013409R	TCTTGTTCCCATTTGACG	The reverse primer for CGI_10013409
Cg-ef1αF	GAGCGTGAACGTGGTATCAC	The forward primer for reference gene
Cg-ef1αR	ACAGCACAGTCAGCCTGTGA	The reverse primer for reference gene

### Data analysis

The responses to the light of the oysters in the experimental and control groups were measured by the relative light-sensitivity rate. The formula is given below. Relative light-sensitivity rate = (number of light-sensitivity after interference/number of illumination after interference)/(number of light-sensitivity before interference/number of illumination before interference). In the RNAi experiment, the responses of the oysters in different groups to the light were compared and tested using independent *t*-test.

## Results

### Vision-related genes were present in the oyster genome and showed higher expression in the mantle

**Figure 1:**
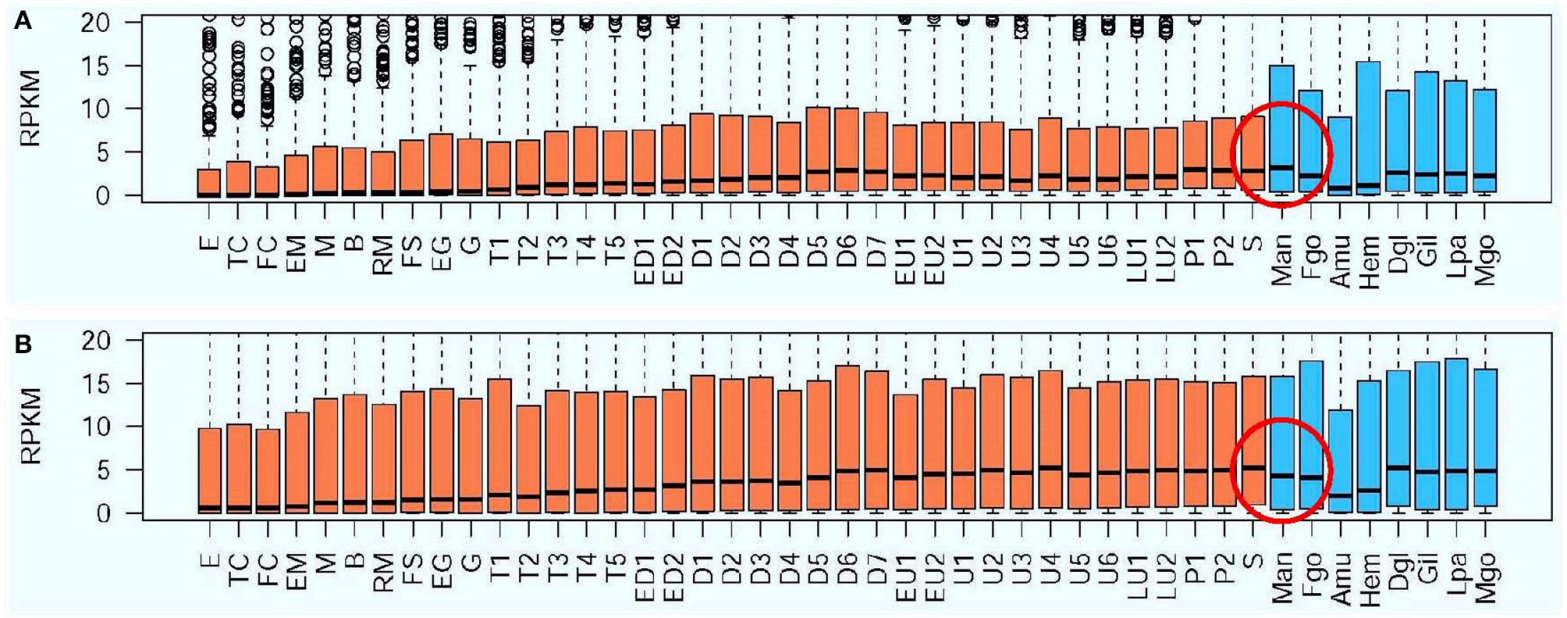
Expression pattern of vision-related genes **(A)** and all other oyster genes **(B)** at different developmental stages and in different organs. Y-axis denotes expression as an RPKM value, while the X-axis denotes the 37 development stages (sienna bars) and the eight organs of adult oyster (steel blue bars). Development stages and organs are abbreviated as follows: E, egg; TC, two cells; FC, four cells; EM, early morula; M, morula; B, blastula; RM, rotary movement; FS, free swimming; EG, early gastrula stage; G, gastrula; T1, trochophore 1; T2, trochophore 2; T3, trochophore 3; T4, trochophore 4; T5, trochophore 5; ED1, early D-larva 1; ED2, early D-larva 2; D1, D-larva 1; D2, D-larva 2; D3, D-larva 3; D4, D-larva 4; D5, D-larva 5; D6, D-larva 6; D7, D-larva 7; EU1, early umbo larva 1; EU2, early umbo larva 2; U1, umbo larva 1; U2, umbo larva 2; U3, umbo larva 3; U4, umbo larva 4; U5, umbo larva 5; U6, umbo larva 6; LU1, later umbo larva 1; LU2, later umbo larva 2; P1, pediveliger 1; P2, pediveliger 2; S, spat; Man, mantle; Fgo, female gonad; Amu, adductor muscle; Hem, haemocyte; Dgl, digestive gland; Gil, gill; Lpa, labial palp; and Mgo, male gonad.

**Figure 2:**
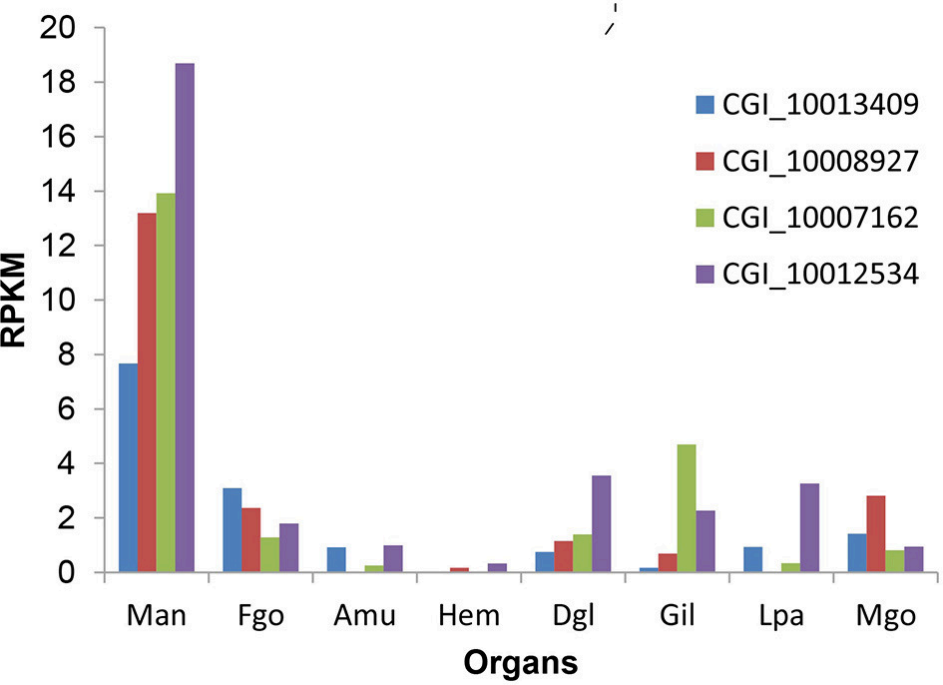
The expression pattern of the four rhodopsin-like superfamily genes. Y-axis denotes expression as an RPKM value and the X-axis indicated different organs. The organs are abbreviated as follows: Man, mantle; Fgo, female gonad; Amu, adductor muscle; Hem, haemocyte; Dgl, digestive gland; Gil, gill; Lpa, labial palp; and Mgo, male gonad.

After scanning the genome of Pacific oyster, 368 vision-related genes were identified, including: rhodopsin-related (Hargrave, 2001; Spudich and Luecke, 2002), opsin-related (Shichida and Matsuyama, 2009; Terakita et al., 2011), melanopsin-related (Hankins et al., 2008; Allen et al., 2017), retinol-related, retinal-related, cones-related (Kawamura and Tachibanaki, 2012), cryptochrome-related (Kawamura and Tachibanaki, 2012), retinoic acid-related (Weiler et al., 1998), retinoid-related, visual perception-related, visual system-related, optic lobes protein-related (Fischbach and Hiesinger, 2008), photoreceptor cell-related (Fain et al., 2010), etc. (Table 3). The detailed gene list is presented in Table S1. Based on the transcriptome of the Pacific oyster (Zhang et al., 2012), the expression pattern of all the vision-related genes at different developmental stages and different organs was constructed. We found that the highest level of gene expression of the vision-related genes was in the mantle, compared to that in other organs (Figure 1A); however, this finding is not true for all oyster genes (Figure 1B). In addition, we found that four rhodopsin-like superfamily genes were specifically expressed in the mantle (Figure 2, the mantle data was the average of in-mantle and out-mantle).

**Table 3:** The vision-related oyster genes in different function categories.

**Gene name**	**Gene number**	**Gene name**	**Gene number**	**Gene name**	**Gene number**	**Gene name**	**Gene number**
Rhodopsin	317	Retinol	18	Optic lobes protein	2	Photoreceptor-specific	1
Regulating retinal	3	Cryptochrome	4	Melanopsin	4	Retinal guanylyl cyclase	1
Opsin	9	Retinoic acid	4	Retinoblastoma	1	Sensitivity to red light	1
Cone photoreceptor	1	Visual perception	3	Photoreceptor cell	2	Retinoids	5
Retinaldehyde-binding	12	Visual system	3	Retinal dehydrogenase	2		

### The siRNA-mediated down-regulation of target genes

**Figure 3:**
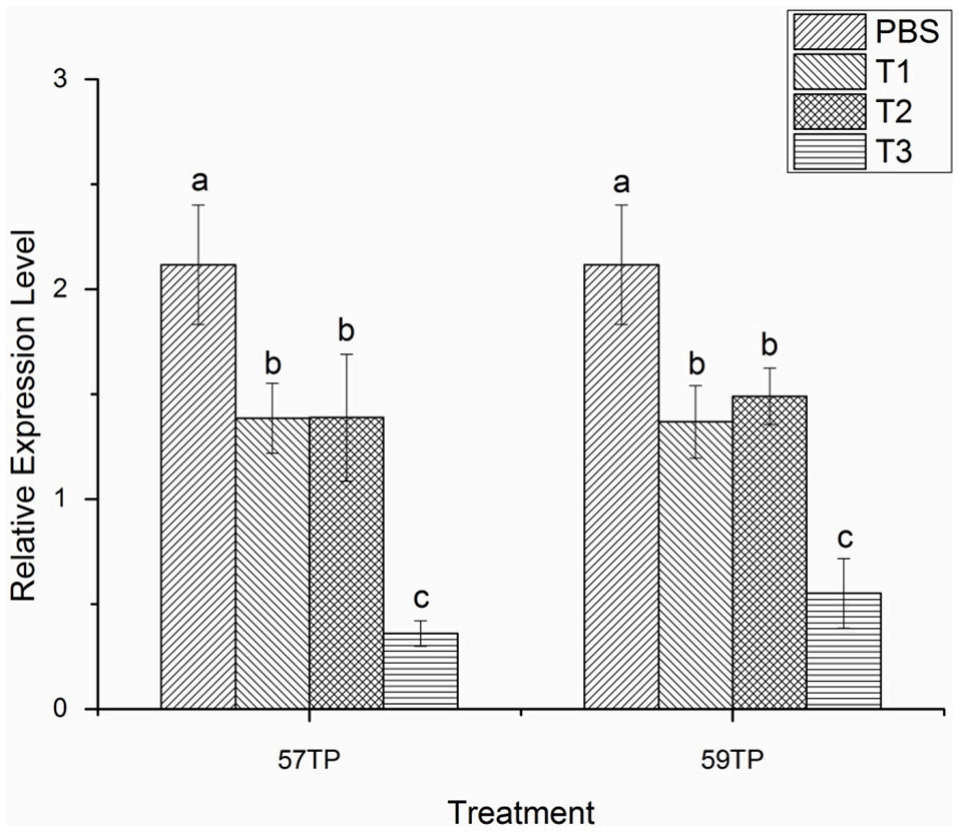
The mRNA expression levels of CGI_10012534 gene in the mantle of oyster after RNAi. Notably, 57TP indicates the siRNA-57 targeted treatment group, 59TP indicates the siRNA-59-targeted treatment group; PBS indicates the group treated with phosphate-buffered saline (PBS) (*n* = 6), T1 indicates the group treated with 5 μg/100 μl siRNA (*n* = 6), T2 indicates the group treated with 10 μg/100 μl siRNA (*n* = 6), and T3 indicates the group treated with 15 μg/100 μl siRNA (*n* = 6). Each bar represents the mean of 6 independent experiments performed in duplicate. Different letters indicate significant difference (*P* < 0.05).

**Figure 4:**
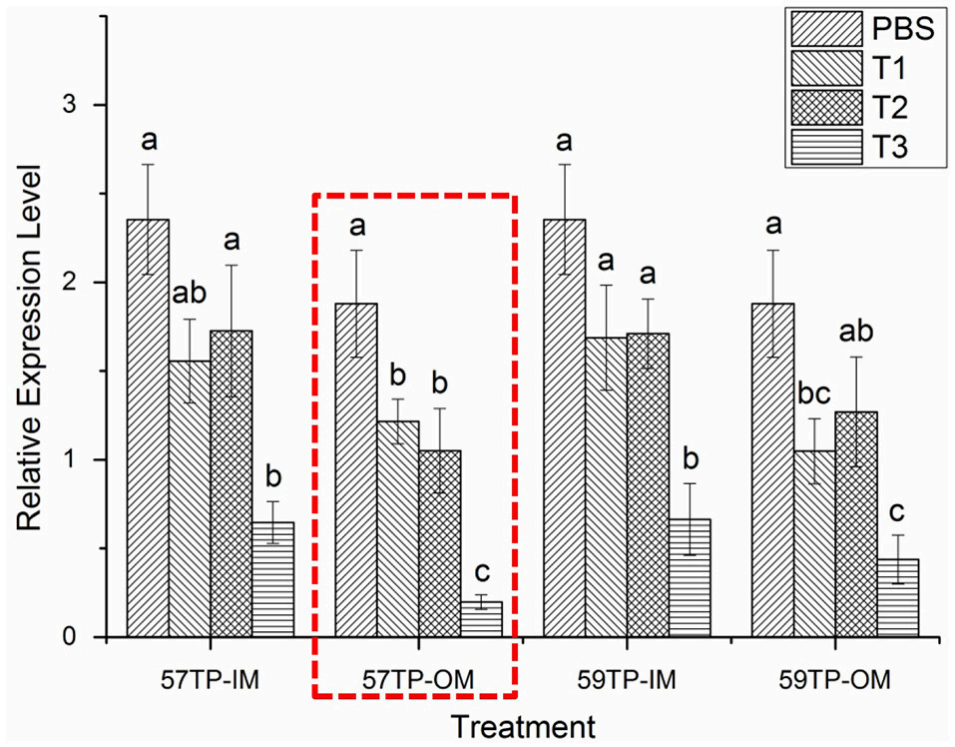
The mRNA expression levels of CGI_10012534 gene in the in-mantle and out-mantle of oysters after RNAi. Notably, 57TP-IM indicates the siRNA-57 targeted treatment group in the in-mantle, 57TP-OM indicates the siRNA-57 targeted treatment group in the out-mantle, 59TP-IM indicates the siRNA-59 targeted treatment group in the in-mantle, 59TP-OM indicates the siRNA-59 targeted treatment group in the out-mantle, PBS indicates the group treated with PBS (*n* = 6), T1 indicates the group treated with 5 μg/100 μl siRNA (*n* = 6), T2 indicates the group treated with 10 μg/100 μl siRNA (*n* = 6), and T3 indicates the group treated with 15 μg/100 μl siRNA (*n* = 6). Each bar represents the mean of six independent experiments. Different letters indicate a significant difference (*P* < 0.05).

The quantitative real-time PCR data were analyzed by the method of 2^−ΔΔ^CT (Livak and Schmittgen, 2001) and showed in Table S2. After the RNA interference experiment, we observed that regardless of the targets, the expression level of the CGI_10012534 gene was lower than that in the control group (PBS), and the expression was significantly lower when the siRNA concentration was 15 μg/100 μl. The mRNA level in the mantle (the data merged from in-mantle and out-mantle) was decreased 1.74 times at the 57TP location and 1.57 times at the 59TP location (Figure 3). Comparison of the data for in-mantle with that for out-mantle revealed a significant gradient of CGI_10012534 gene expression in the out-mantle with an increasing dsRNA concentration, and the interference effect was significant at a concentration of 15 μg/100 μl (Figure 4, in the red frame; *P* < 0.05).

However, the other three genes (CGI_10013409, CGI_10008927 and CGI_10007162) did not show any significant differences (*P* > 0.05) or gradient differences (Figures S1–S3). Thus, no further analysis or experiments were performed for these genes.

### The light-sensitivity before and after RNAi

**Figure 5:**
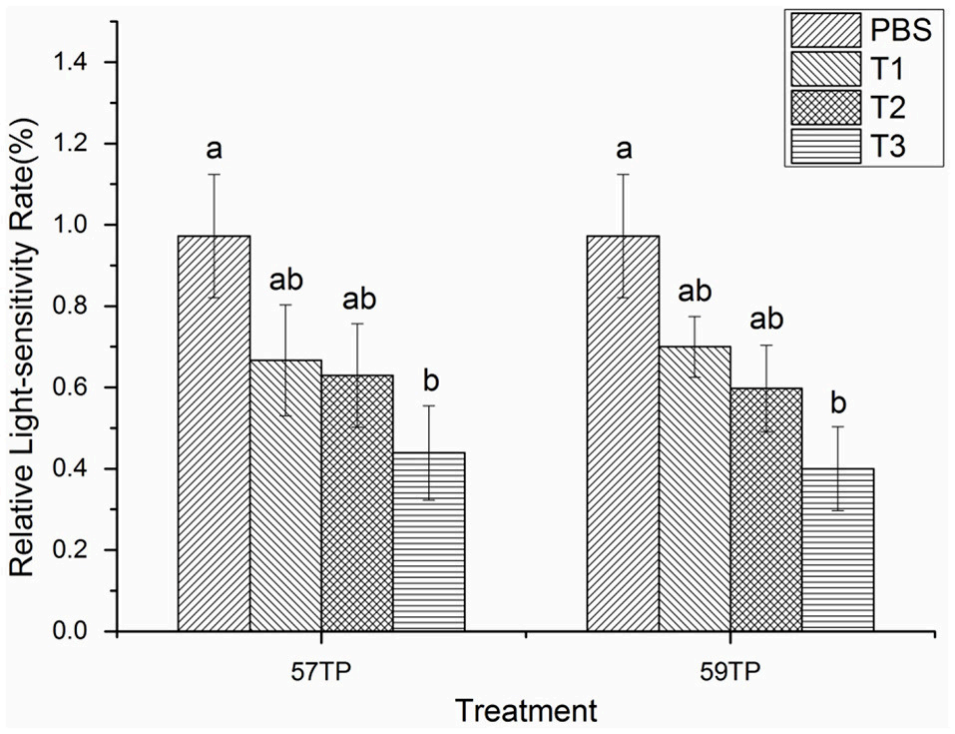
Light-sensitivity changes before and after the RNAi interference of the CGI_10012534 gene. Notably, 57TP indicates the siRNA-57 targeted treatment group and 59TP indicates the siRNA-59 targeted treatment group. PBS indicates the group treated with PBS (*n* = 6), T1 indicates the group treated with 5 μg/100 μl siRNA (*n* = 6), T2 indicates the group with 10 μg/100 μl siRNA (*n* = 6), and T3 indicates the group with 15 μg/100 μl siRNA (*n* = 6). Each bar represents the mean of six independent experiments. Different letters indicate a significant difference (*P* < 0.05).

Comparison of the light response of the oyster before and after RNA interference of the CGI_10012534 gene revealed that the light-sensitivity of the oyster was generally decreased with an increasing dsRNA concentration, although the differences were not significant (*P* < 0.05) at concentrations of 5 μg/100 μl and 10 μg/100 μl. In particular, the light-sensitivity of oysters was significantly reduced (*P* < 0.05) at a concentration of 15 μg/100 μl not only in the 57-TP but also 59-TP treatment groups (Figure 5), compared with the PBS group. The reduction tendency of light response was consistent with the decreasing tendency of mRNA expression when the dose of dsRNA was raised gradually.

## Discussion

It has been previously assumed that the adult oyster did not need the ability to sense light, because oysters have a sessile lifestyle after larva settlement and metamorphosis (Zhang et al., 2012). Nevertheless, when the oyster filters seawater to obtain food and oxygen, its shells must be opened. During this period, the oyster may be vulnerable to attack by predators. In this context, the perception of light change could be very important to avoid being preyed, which may be reason why oysters evolved the ability of light-sensitivity. In our previous experiment, it has been observed that adult Pacific oyster has the ability of light-sensitivity (Wu et al., 2015).

Rhodopsin is the largest class of G protein coupled receptors (family A, subsequently referred to as GPCR-A; Bryson-Richardson et al., 2004) and a major component of rod photoreceptors; this receptor is photosensitive, even under extremely weak light conditions (Hargrave and McDowell, 1992). So far, there are no reports on the oyster rhodopsin superfamily genes. In the present study, we first found that the expression of four rhodopsin genes in the mantle was significantly higher than that in the other organs. CGI_1001253 gene is one of four rhodopsin-like superfamily members.

RNAi and light-sensitivity detection experiments revealed significant differences in the light-sensitivity of the oysters between the control and treatment groups, and increasing the concentration of injected dsRNA progressively decreased the mRNA expression level of the CGI_10012534 gene; importantly, the light-sensitivity of the corresponding oysters was also gradually weakened. These results may suggest that the CGI_10012534 gene is associated with light-sensitivity in the adult Pacific oyster.

Interestingly, there is a strict linear relationship between the mRNA expression level of CGI_10012534 gene and the light-sensitivity of the corresponding oysters in the out-mantle (57TP-OM), but not in the in-mantle, suggesting that the out-mantle may play a more important role in light-sensitivity than the in-mantle.

The eye was defined as any organ with the necessary components for rudimentary spatial resolution or image-forming capabilities. There are distinct eye types in molluscs, from the pit eyes of many gastropods, to the pinhole eyes of the Nautilus, to the lensed eyes of the cephalopods. Compound eyes are present in some bivalves, and reflective “mirrors” have been innovated by other lineages such as scallops (Serb and Eernisse, 2008). In the present study, we could not determine whether the eye exists in the oyster; however, we obtained preliminary evidence suggesting that the CGI_10012534 gene may be one of the genes associated with light-sensitivity. Thus, more direct evidence should be obtained through transgenic methods (such as the CRISPR method) in the future.

In addition, in the present study, we attempted to determine whether the oyster had a reaction to light changes by the naked eye; however, some behavioral changes could not be observed if the valve opening magnitude was too small. Recently, high-frequency non-invasive valvometry technology was used to record the valve movements for measuring the sensitivity of the oysters (Charifi et al., 2017). In future, we will find the fitting point that can combine the high-frequency non-invasive valvometry with the study of oyster light sensitivity.

## Author contributions

XW conceived and designed the experiments; CW, QJ, LW, JC, and ZC performed the experiments; WY, CH, and JW analyzed the data; ZC and WG contributed reagents, materials, analysis tools; XW, CW, and QJ wrote the paper. All authors reviewed the manuscript.

### Conflict of interest statement

The authors declare that the research was conducted in the absence of any commercial or financial relationships that could be construed as a potential conflict of interest.

## Supplementary material

Figure S1 The mRNA expression levels of CGI_10013409 gene in the mantle of oyster after RNAi. Notably, 99TP indicates the siRNA-99 target treatment group, 76TP indicates the siRNA-76 target treatment group; PBS indicates the group treated with phosphate-buffered saline (PBS), T1 indicates the group treated with 5 μg/100 μl siRNA, T2 indicates with 10 μg/100 μl siRNA, and T3 indicates the group treated with 15 μg/100 μl siRNA. Each bar represents the mean of six independent experiments performed in duplicate. Different letters indicate a significant difference (*P* < 0.05).

Figure S2 The mRNA expression levels of CGI_10008927 gene in the mantle of oyster after RNAi. Notably, 31TP indicates the siRNA-31 target treatment group, 43TP indicates the siRNA-43 target treatment group; PBS indicates the group treated with phosphate-buffered saline (PBS), T1 indicates the group treated with 5 μg/100 μl siRNA, T2 indicates the group treated with 10 μg/100 μl siRNA, and T3 indicates the group treated with 15 μg/100 μl siRNA. Each bar represents the mean of six independent experiments performed in duplicate. Different letters indicate a significant difference (*P* < 0.05).

Figure S3 The mRNA expression levels of CGI_10007162 gene in the mantle of oyster after RNAi. Notably, 539TP indicates the siRNA-539 target treatment group, 757TP indicates the siRNA-757 target treatment group; PBS indicates the group treated with phosphate-buffered saline (PBS), T1 indicates the group treated with 5 μg/100 μl siRNA, T2 indicates the group treated with 10 μg/100 μl siRNA, and T3 indicates the group treated with 15 μg/100 μl siRNA. Each bar represents the mean of six independent experiments performed in duplicate. Different letters indicate a significant difference (*P* < 0.05).

Table S1 Vision-related genes in different categories.

Table S2 The data for mRNA expression levels of CGI_1001253 gene.
