# Supplementary material for: A Rhodopsin-Like Gene May Be Associated With the Light-Sensitivity of Adult Pacific Oyster Crassostrea gigas
Source: Front Physiol. 2018 Mar 19;9:221. doi: 10.3389/fphys.2018.00221 (PMC5868073; doi:10.3389/fphys.2018.00221)
Supplement: Figure S1 — The mRNA expression levels of CGI_10013409 gene in the mantle of oyster after RNAi. Notably, 99TP indicates the siRNA-99 target treatment group, 76TP indicates the siRNA-76 target treatment group; PBS indicates the group treated with phosphate-buffered saline (PBS), T1 indicates the group treated with 5 μg/100 μl siRNA, T2 indicates with 10 μg/100 μl siRNA, and T3 indicates the group treated with 15 μg/100 μl siRNA. Each bar represents the mean of six independent experiments performed in duplicate. Different letters indicate a significant difference (P < 0.05). [file Image1.PDF]

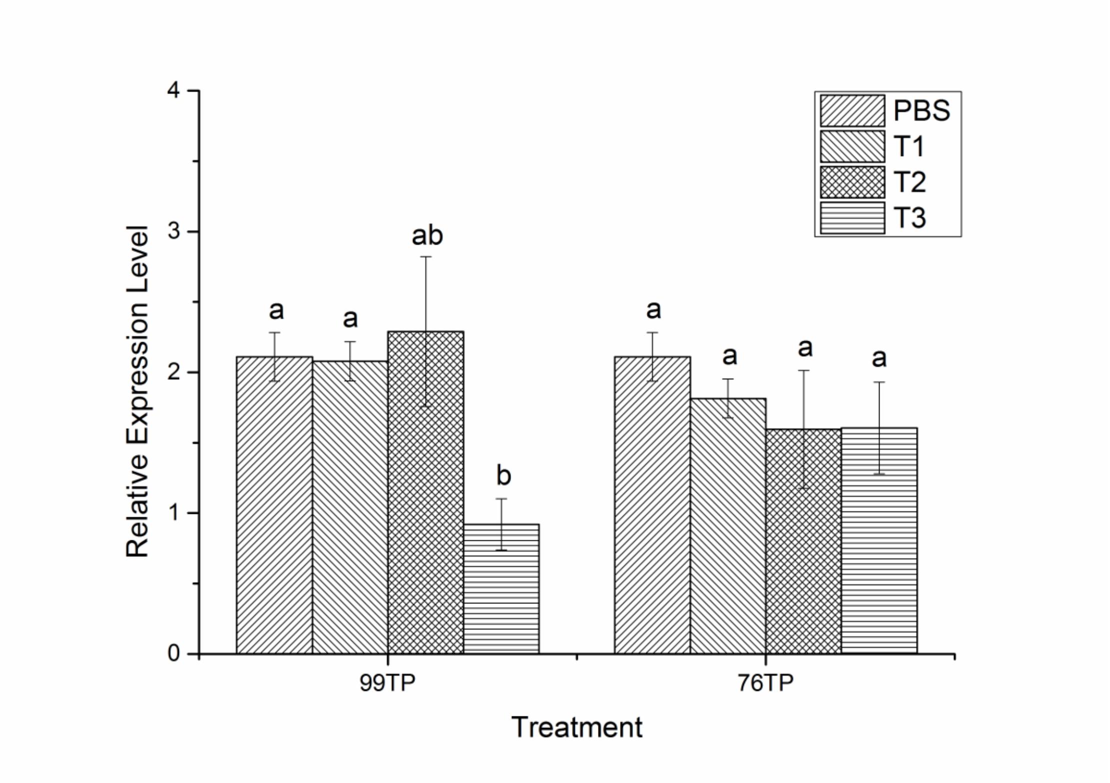

Figure S1. The mRNA expression levels of CGI_10013409 gene in the mantle of oyster after RNAi. Notably, 99TP indicates the siRNA-99 target treatment group, 76TP indicates the siRNA-76 target treatment group; PBS indicates the group treated with phosphate-buffered saline (PBS), T1 indicates the group treated with 5 μg / 100 μl siRNA, T2 indicates with 10 μg / 100 μl siRNA, and T3 indicates the group treated with 15 μg / 100 μl siRNA. Each bar represents the mean of 6 independent experiments performed in duplicate. Different letters indicate a significant difference ($P < 0.05$).
